# Gating Techniques for Rao-Blackwellized Monte Carlo Data Association Filter

**DOI:** 10.1155/2014/960857

**Published:** 2014-03-03

**Authors:** Yazhao Wang, Peini Zhang

**Affiliations:** ^1^The Department of Systems and Control, Beihang University (BUAA), Beijing 100191, China; ^2^School of Mechanical Electronic and Information Engineering, China University of Mining and Technology, Beijing 100083, China

## Abstract

This paper studies the Rao-Blackwellized Monte Carlo data association (RBMCDA) filter for multiple target tracking. The elliptical gating strategies are redesigned and incorporated into the framework of the RBMCDA filter. The obvious benefit is the reduction of the time cost because the data association procedure can be carried out with less validated measurements. In addition, the overlapped parts of the neighboring validation regions are divided into several separated subregions according to the possible origins of the validated measurements. In these subregions, the measurement uncertainties can be taken into account more reasonably than those of the simple elliptical gate. This would help to achieve higher tracking ability of the RBMCDA algorithm by a better association prior approximation. Simulation results are provided to show the effectiveness of the proposed gating techniques.

## 1. Introduction

Data association plays an important role in filtering methods for multitarget tracking in cluttered (or false alarm) environment. Many approaches have been developed to solve this problem [2–7]. One solution is the Rao-Blackwellized Monte Carlo data association (RBMCDA) [1, 8]. In this approach, particle filter (PF) is only used to evaluate the data association indicators instead of computing everything by pure Monte Carlo sampling. Therefore, the required number of particles can be significantly reduced. This can be a method with good performance for tracking a single target or multiple separated targets in sparsely cluttered environment. Unfortunately, we found in experiments that the time cost of this algorithm is too large even in modest clutter environment. Besides, it will lead to significantly low tracking performance of the algorithm when several targets as well as clutter are present in the same neighborhood. To address these problems, this paper employs gating technique for improvement.

In traditional tracking algorithms, a gate (validation region) can be used to guarantee that the target originated measurement falls into it with high (gate) probability [2–4]. The probabilistic data association (PDA) [4] has been thought to be a very good elliptical gate based method for tracking a single target or several isolated targets even in dense cluttered environment. On this basis, many types of gating techniques have been studied [9–12]. Specially, the Voronoi measure is used to select validated measurements for single target tracking in [13]. When several targets are present in the same neighborhood in addition to the random interference from clutter, a more complicated situation should be considered. The joint probabilistic data association (JPDA) can give a joint probability by first enumerating the joint events [3, 14]. In JPDA, not only the number of the validated measurements can be reduced to a low level, but also the problem of measurement uncertainty can be addressed very well. Because enumerating the joint association events is often computationally intensive [15], many time saving suboptimal JPDA filtering methods are available, such as Fitzgerald's and other ad hoc implementations [2, 16, 17]. Recently, the elliptical gating technique has also been studied and redesigned for reducing the computational cost of the popular Gaussian mixture- cardinalized probability hypothesis density (GM-CPHD) filter [18, 19].

The remainder of this paper is organized as follows.  Section 2 gives a brief introduction on the RBMCDA filter and the validation region.  Section 3 presents three different gating strategies according to the framework of the RBMCDA filter. Numerical examples and simulation results are presented in Section 4, followed by conclusions in Section 5.

## 2. Preliminaries

### 2.1. Rao-Blackwellized Monte Carlo Data Association Filter

Consider the following time-varying system:

(1) $$\begin{array}{c} x_{k}^{1}=F_{k-1}x_{k-1}^{1}+w_{k-1},\\ z_{k}=H_{k}x_{k}^{1}+v_{k}, \end{array}$$

where **x**_*k*_^1^ and **z**_*k*_ are the state and measurement variables in time step *k*, respectively. **w**_*k*−1_ ~ *𝒩*(0, **Q**_*k*−1_) and **v**_*k*_ ~ *𝒩*(0, **R**_*k*_) are zero mean mutually independent Gaussian noise and **F**_*k*−1_ and **H**_*k*_ are with compatible dimensions and represent the state and measurement transition matrices, respectively. Suppose that we are able to form another variable **x**_*k*_^2^ to describe these matrices, the so-called Rao-Blackwellized particle filter (RBPF) algorithm can be applied to estimate the whole state **x**_*k*_ = {**x**_*k*_^1^; **x**_*k*_^2^} [20]. For space consideration, we omit the details of the RBPF algorithm which can be found in [21–24]. For this conditionally linear-Gaussian system, the Kalman series filter prediction and update steps can be performed for each target in each of the particles separately.

In [1], it has been shown that the RBMCDA algorithm can be obtained directly from the above RBPF framework when the latent value **x**_*k*_^2^ is defined to be the data association event indicators *c*_*k*_,

(2) $$\begin{array}{c} x_{k}^{2}=c_{k}, \end{array}$$

where the value *c*_*k*_ = 0 when the measurement is from clutter and *c*_*k*_ = *j* when the measurement is from target *j*. The predictive probability *p*(*c*_*k*_ | *c*_1:*k*−1_) gives the priors of data association given the data association results {*c*_1:*k*−1_} in the *k* − 1 previous time steps. The calculation of the *p*(*c*_*k*_ | *c*_1:*k*−1_, **z**_1:*k*_) (posterior distribution of *c*_*k*_) is given by

(3) $$\begin{array}{c} p\left(c_{k}\;|\;c_{1:k-1},z_{1:k}\right)\propto p\left(z_{k}\;|\;c_{k},c_{1:k-1},z_{1:k-1}\right)p\left(c_{k}\;|\;c_{1:k-1}\right). \end{array}$$

The main difficulty encountered in RBMCDA is the computation of the association prior probabilities *p*(*c*_*k*_ | *c*_1:*k*−1_). In [1], the data association priors are modeled as a recursive Markov chain to make sure of one target per measurement in the same time steps. Accordingly, the *p*(*c*_*k*_ | *c*_1:*k*−1_) in (3) has been replaced by the *p*(*c*_*k*_ | *c*_*k*−*M*:*k*−1_) and

(4) $$\begin{array}{c} p\left(c_{k}\;|\;c_{1:k-1},z_{1:k}\right)\propto p\left(z_{k}\;|\;c_{k},c_{1:k-1},z_{1:k-1}\right)p\left(c_{k}\;|\;c_{k-M:k-1}\right). \end{array}$$

The general form of the joint prior model is given by [1]

(5) $$\begin{array}{c} p\left(c_{k+M-1},\ldots ,c_{k}\right)=\prod_{m=1}^{M}p\left(c_{k+m}\;|\;c_{k},\ldots ,c_{k+m-1}\right). \end{array}$$

That means if we obtain *M* measurements in time step *k*, the calculation of the *m*th (*m* = 2,…, *M*) measurement's association prior *p*(*c*_*k*+*m*−1_ | *c*_*k*+*m*−2_,…, *c*_*k*_) only depends on the *m* − 1 previous associations {*c*_*k*+*m*−2_,…, *c*_*k*_} in the *k*th time step (note that, if *m* = 1, *c*_*k*_ has the prior *p*(*c*_*k*_)). Assume we track *T* targets; the detection indicator for the *j*th (*j* = 1,…, *T*) target can be given by *δ*_*j*_(*m* − 1). It is a binary indicator to indicate that if the *j*th target has been assigned a measurement in {*c*_*k*+*m*−2_,…, *c*_*k*_}, the detection indicator combinations *δ*_1:*T*_(*m* − 1) decide the data association priors as

(6) $$\begin{array}{c} p\left(c_{k+m-1}\;|\;\delta_{1:T}\left(m-1\right)\right)=f_{c}\left(P_{d}^{\left(j\right)}\right), \end{array}$$

where *f*_*c*_ is a function with definite forms corresponding to the results of *δ*_1:*T*_(*m* − 1) [1]. We can see that only the target detection probability *P*_*d*_^(*j*)^ (in (6)) needs to be given at the beginning of the Markov recursive calculation procedures. Since *P*_*d*_^(*j*)^ is assumed to be a known value [1], data association (the posterior distribution *p*(*c*_*k*_ | *c*_1:*k*−1_, **z**_1:*k*_)) is in fact totally determined by the measurement likelihood *p*(**z**_*k*_ | *c*_*k*_, *c*_1:*k*−1_, **z**_1:*k*−1_) according to (4), (5), and (6).

### 2.2. Validation Region

A gate is set up for selecting the measurement originated from the target in high probability, and gate can also be called gating region or validation region. In practical tracking algorithms, validation region is often used to reduce the cardinality of measurement set. Measurements outside the validation region can be ignored reasonably because the probabilities of them being from the corresponding targets are quite low according to the statistical characterization. Data association method often incorporates an elliptical validation region [3] as

(7) $$\begin{array}{c} \Gamma_{k}\left(\gamma \right)=\left\{z_{k}:{\left[z_{k}-{\hat{z}}_{k\;|\;k-1}\right]}^{\prime }S{\left(k\right)}^{-1}\left[z_{k}-{\hat{z}}_{k|k-1}\right]\leq \gamma \right\}, \end{array}$$

where *γ* is the gate threshold corresponding to the gate probability *P*_*g*_ which is the probability that the gate contains the true measurement and **S**(*k*) is the covariance of the innovation corresponding to the true measurement **z**_*k*_. In this paper, we just consider the elliptical gating technique. For each target (*j* = 1,2,…, *T*), the gate probability *P*_*g*_^(*j*)^ = 0.9997 with *γ* = 16 [3].

**Figure 1:**
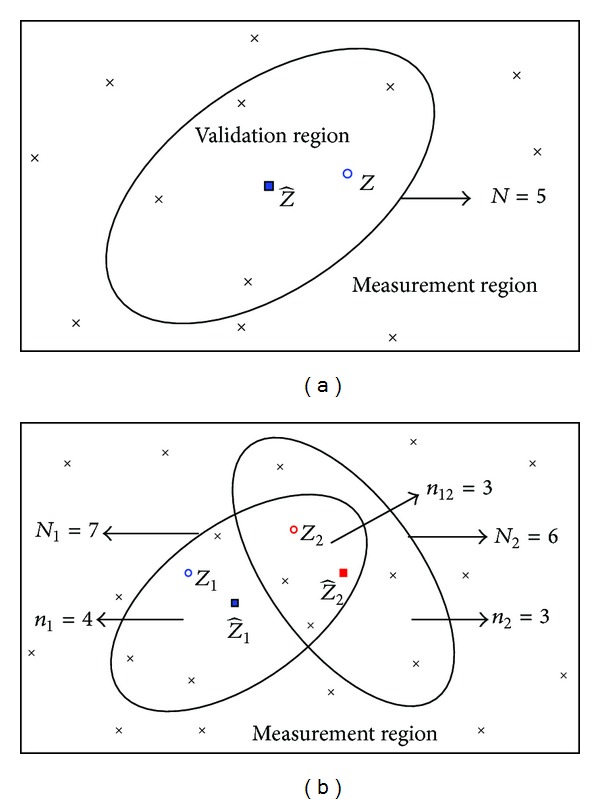
Examples of measurement validation region: (a) for a single target, (b) for two targets.

Figure 1(a) gives an example of a single target's measurement validation region. *N* = 5 represents the number of validated measurements in this elliptical region. For this 2*D* example, a measurement is considered to be validated for target *j* if it falls inside the elliptical region centered at the predicted measurement $\hat{Z}$ (“■” represents the location of $\hat{Z}$). The target originated measurement *Z* (“∘”) and clutter originated measurement (“×”) are represented by the different symbols, but they actually show no difference from a tracker's perspective in applications. Thus, each one of the five measurements in the validation region can be originated from the target or clutter.

Figure 1(b) illustrates a case of two targets as well as clutter being present in the same neighborhood. The predicted measurements for target 1 and target 2 are denoted by ${\hat{Z}}_{1}$ and ${\hat{Z}}_{2}$, respectively. There are three measurements in the intersection of their validation regions (*n*_12_ = 3); *n*_1_ = 4 and *n*_2_ = 3 are the number of the validated measurements in the isolated parts of the two elliptical validation regions. *N*_1_ = 7 and *N*_2_ = 6 represent the numbers of validated measurements in their elliptical regions, respectively. This example shows the interdependence of measurement origins. The RBMCDA, like all other data association methods, has also to deal with these uncertainties.

### 2.3. Problem Statement

In the original RBMCDA approaches [1, 8], all received measurements in the entire measurement space are considered as the validated measurements. From the tracker's point of view, the target detection probability *P*_*d*_^(*j*)^ mentioned in Section 2.1 represents the probability that the target-originated measurement falls into the whole measurement space. When a gating technique is considered, the data association is restricted into the validation regions so that the *P*_*d*_^(*j*)^ used in (6) should be replaced by *P*_*e*_^(*j*)^ accordingly. That is

(8) $$\begin{array}{c} p\left(c_{k+m-1}\;|\;\delta_{1:T}\left(m-1\right)\right)=f_{c}\left(P_{e}^{\left(j\right)}\right), \end{array}$$

where *P*_*e*_^(*j*)^ defines the probability that the target-originated measurement falls into a concerned (validation) region. There is no doubt that the number of the validated measurements will be reduced to a low level followed by the possible reduction of the running time [19]. Consequently, the key problem becomes the calculation of *P*_*e*_^(*j*)^.

## 3. Gating Techniques for RBMCDA Filter

In this section, three different gating techniques are presented applicable to the framework of the RBMCDA filter, and the required *P*_*e*_^(*j*)^ is discussed in detail. A simple gating method is first discussed. We then present the joint events (J-) based gating method which takes into account the interference from the neighboring targets. To get a practical solution with low time cost, then a simplified version of the “J-” method (SJ-) is proposed. At last, the main procedure of the gating technique based RBMCDA algorithm is given for clarity.

### 3.1. The Union Based Method

**Figure 2:**
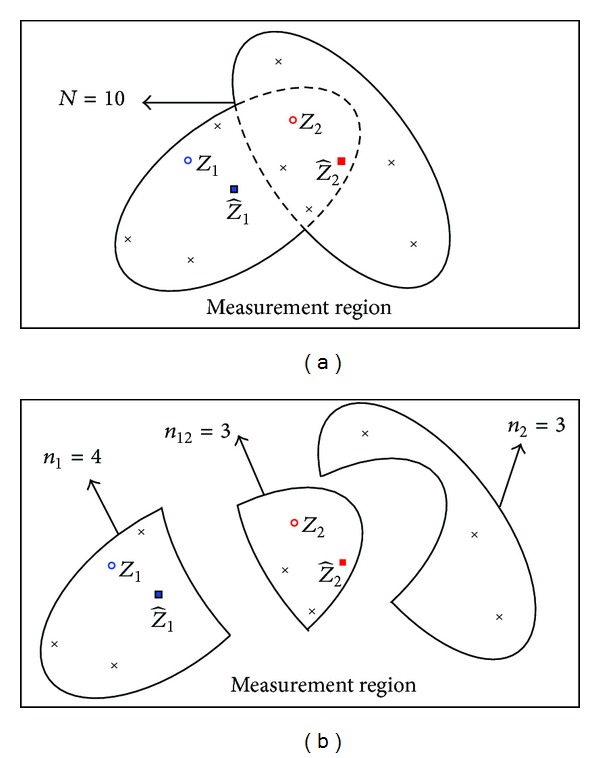
Regulations of measurement validation regions, (a) as a union, (b) in separated forms.

To reduce the cardinality of the validated measurement set, we can define a union which consists of all the elliptical validation regions (see Figure 2(a)). In this example, only the validated measurements in the defined union are depicted. The dotted lines represent the boundaries of the common region of the two elliptical validation regions. This union based RBMCDA algorithm is termed U-RBMCDA for short. In this method, the boundaries of the common region can be omitted and each measurement falling inside the union is assumed to be the validated measurement of both the two targets. Hence, the required *P*_*e*_^(*j*)^ can be obtained directly by

(9) $$\begin{array}{c} P_{e}^{\left(j\right)}=P_{d}^{\left(j\right)}P_{g}^{\left(j\right)}. \end{array}$$

We can see that, in this union, the *P*_*e*_^(*j*)^ will be a fixed value if *P*_*d*_^(*j*)^ and *P*_*g*_^(*j*)^ are known. The difference between U-RBMCDA and the O-RBMCDA (the original RBMCDA [8]) algorithm is that the former just reserves the measurements within the union as the validated measurements for all targets. However, it is not difficult to find that the validated measurements outside the common region could be possibly originated from only one target according to their locations. For example, as shown in Figure 1(b), the four measurements (*n*_1_ = 4) in the left part of the 1st target's region could only be possibly from target 1. That is to say, the union is too big to differentiate the measurements with different possible origins. To address this, the detailed measurement location information is needed to consider, and it will go more reasonable if we get the estimates of the target existent probabilities in the smaller regions.

In fact, the joint validation region can be seen as consisting of several sub-validation-regions (SVRs) according to the possible origins of the validated measurements, and the *P*_*e*_^(*j*)^ for a target *j* could have different values in different subregions. Therefore, we should divide the joint validation region into several SVRs to make sure the validated measurements within a SVR are with the same possible origins. To emphasize this point, Figure 2(b) gives a separated but equivalent form of illustration as that of Figure 1(b). In this example, the numbers of validated measurements in three SVRs are *n*_1_ = 4, *n*_2_ = 3, and *n*_12_ = 3, respectively.

### 3.2. Joint Events Based Gating Method

In a SVR with identifier *r*, we need to calculate the probability *P*_*e*_*r*__^(*j*)^ for the *j*th (*j* = 1,2,…, *T*) target. If we still want to calculate *P*_*e*_*r*__^(*j*)^ by the way of *P*_*e*_*r*__^(*j*)^ = *P*_*g*_*r*__^(*j*)^ *P*_*d*_^(*j*)^ (*P*_*d*_*r*__^(*j*)^ = *P*_*d*_^(*j*)^), the target gate probability *P*_*g*_*r*__^(*j*)^ should be known. Although the *P*_*g*_^(*j*)^ can be directly obtained according to (7), it is hardly possible to obtain the analytical solutions of the gate probability *P*_*g*_*r*__^(*j*)^ in an arbitrary SVR with identifier *r*. That is because a SVR is usually one part of the ellipse or the other irregular shape (see Figure 2(b)). In this case, the required measurements statistical information which determines the *P*_*g*_*r*__^(*j*)^ will be extremely difficult to obtain. To solve this problem, we present the joint events based method and use the weights of the probabilities of the feasible events instead.

In this process, the possible events can be constructed in a similar way as JPDA does [3, 17]. To build the joint events and derive the joint probabilities, the key information required is the numbers of the measurements in these SVRs. In fact, the validated measurements in different SVRs can be differentiated by using the distance measure from the center points of the corresponding validation regions (see (7)), so that the required measurements numbers are not difficult to obtain. The probability of an event *θ*_*k*_ for all *M* validated measurements in the joint validation region is given by

(10) P{θk}=P{θk,δ,nc}=P{θk ∣ δ,nc}P{δ,nc}=nc!M!∏m=1M(Pd(j))δj(1−Pd(j))1−δjfp(nc),

where *δ* indicates from which target the present measurement is originated, while *δ*_*j*_ (*δ*_*j*_ = 1 or 0) indicates that whether the *m*th measurement has been considered to be originated from the *j*th target. Both {*θ*_*k*_} and *δ*_*j*_ are determined by the numbers of the validated measurements in the SVRs. *P*_*d*_^(*j*)^ is the detection probability for the *j*th target, *n*_*c*_ is the number of the measurements considered to be form clutter, and *f*_*p*_(*n*_*c*_) represents the nonparametric prior probability of the number of clutter originated measurements in the joint validation region. Note that the *f*_*p*_(*n*_*c*_) and *M*! can be canceled in a normalizing factor *α* [3] as

(11) $$\begin{array}{c} P\left\{\theta_{k}\right\}=\frac{n_{c}!}{\alpha }\prod_{m=1}^{M}{\left(P_{d}^{\left(j\right)}\right)}^{\delta_{j}}{\left(1-P_{d}^{\left(j\right)}\right)}^{1-\delta_{j}}. \end{array}$$

The weighting factor *P*_*k*_^(*mj*)^ represents the probability of the *m*th measurement being originated from target *j* and it is given by

(12) $$\begin{array}{c} P_{k}^{\left(mj\right)}=\sum_{\theta_{k}}p\left(\theta_{k}\right)\beta \left(\theta_{k}\right), \end{array}$$

where *β*(*θ*_*k*_) is a binary variable which indicates whether the *m*th measurement is possibly originated from the *j*th target. In following discussions, the identifier and *k* can be omitted without misunderstanding and *P*_*k*_^(*mj*)^ can be rewritten as *P* ^(*mj*)^. In a SVR with identifier *r*, *P*_*e*_*r*__^(*j*)^ is the sum of the weighting factor *P* ^(*mj*)^

(13) $$\begin{array}{c} P_{e_{r}}^{\left(j\right)}=\sum_{m=m_{1}}^{m_{r}}P^{\left(mj\right)}, \end{array}$$

where {*m*_1_,…, *m*_*r*_} is set of the measurements in the *r*th SVR. If *P*_*e*_*r*__^(*j*)^ = 1, the *j*th target originated (true) measurement is certainly in the *r*th SVR.

### 3.3. Simplified Joint Events Based Gating Method

To develop a practical suboptimal J-RBMCDA method with less time cost, we present a simplified joint events (SJ-) based gating technique. In J-RBMCDA, the numbers of validated measurements in the SVRs play the key roles to calculate the target existent probability or even determine the results. In fact, they represent the relative importance of these subregions, or the gate weights of the SVRs. So we can use the normalized weights and the target detection probability *P*_*d*_^(*j*)^ to estimate *P*_*e*_^(*j*)^. In each time step *k*, the computational formulas are as follows:

(14) $$\begin{array}{c} N_{j}=n_{j}+\sum_{C_{1}}n_{jk_{1}}+\cdots +\sum_{C_{T-1}}n_{jk_{1}k_{2}\cdots k_{T-1}},\\ P_{j}^{\left(j\right)}=\frac{n_{j}}{N_{j}};\\ P_{jk_{1}}^{\left(j\right)}=\frac{n_{jk_{1}}}{N_{j}},\;k_{1}\in C_{1};\ldots ;\\ P_{jk_{1}k_{2}\cdots k_{T-1}}^{\left(j\right)}=\frac{n_{jk_{1}k_{2}\cdots k_{T-1}}}{N_{j}},\;\left\{k_{1}k_{2}\cdots k_{T-1}\right\}\in C_{T-1}. \end{array}$$

In (14), *N*_*j*_ is the number of validated measurements in the elliptical validation region of the *j*th (*j* = 1,2,…, *T*) target and *n*_*jk*_1_*k*_2_⋯*k*_*t*__ (*t* = 1,2,…, *T* − 1) is the number of validated measurements in the SVR with identifier *jk*_1_ *k*_2_ ⋯ *k*_*t*_. Moreover, *C*_*t*_ defines a SVR set in which each element is related to the *j*th target itself and other *t* targets:

(15) C1={k1:1≤k1≤T, k1≠j};…;CT−1={(k1k2⋯kT−1):k1,k2,…,kT−1  are  (T−1)   different numbers in{1,2,…,T}∖{j}}.

As a result, *P*_*jk*_1_*k*_2_⋯*k*_*t*__^(*j*)^ is the estimated gate weight of the *j*th target in the *jk*_1_ *k*_2_ ⋯ *k*_*t*_th SVR. Since the target gate probability *P*_*g*_^(*j*)^ in the *j*th elliptical region and the target detection probability *P*_*d*_^(*j*)^ have been assumed to be known, in a SVR with identifier *jk*_1_ *k*_2_ ⋯ *k*_*t*_, the *P*_*e*_*jk*_1_*k*_2_⋯*k*_*t*___^(*j*)^ can be given by

(16) $$\begin{array}{c} P_{e_{jk_{1}k_{2}\cdots k_{t}}}^{\left(j\right)}=P_{jk_{1}k_{2}\cdots k_{t}}^{\left(j\right)}P_{g}^{\left(j\right)}P_{d}^{\left(j\right)}. \end{array}$$

Note that the SVR identifier (*jk*_1_ *k*_2_ ⋯ *k*_*t*_) is independent of the order of *j*, *k*_1_, *k*_2_,…, *k*_*t*_. For example, when *j* = 1 and *k*_1_ = 2, there are *n*_12_ = *n*_21_ and *P*_*e*_12__^(1)^ = *P*_*e*_21__^(1)^. However, in the SVR with the identifier 12 (or 21), *P*_*e*_12__^(1)^ = (*n*_12_/*N*_1_)*P*_*d*_^(1)^ and *P*_*e*_12__^(2)^ = (*n*_12_/*N*_2_)*P*_*d*_^(2)^ are different values for target 1 and target 2, respectively. Hence, even in the same SVR, *P*_*e*_*r*__^(1)^ and *P*_*e*_*r*__^(2)^ have different values.

This simplified joint events based RBMCDA (SJ-RBMCDA) can be seen as a very simple form of the J-RBMCDA with the joint events being broken up into several isolated events. The significant difference is that, in SJ-RBMCDA, the procedure of enumerating the possible association events is replaced by a simple nonparametric method. Therefore, the required *P*_*e*_*r*__^(*j*)^ can be computed by directly using the numbers of the validated measurements in the corresponding SVRs. Logically, less computational time is needed.

**Figure 3:**
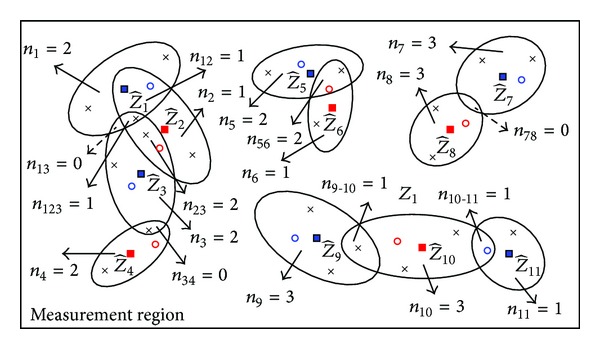
An example of measurement validation regions for a large number of targets.

It should be pointed out that the J-RBMCDA and SJ-RBMCDA algorithms may provide more weights to the right SVRs if the tracker can get the correct target estimates. Like all other gating based algorithms, however, the performance of the proposed gating based RBMCDA algorithms will also be degraded in the cases that the tracker provides incorrect target estimates. Another problem is that, for both J-RBMCDA and SJ-RBMCDA, the number of SVRs seems to increase dramatically with the number of targets to be tracked.  Figure 3 gives an example of tracking 11 targets, and each of the arrows points to a SVR. We can see that the total number of the SVRs is 20 including the empty SVRs (pointed by dotted arrows), so that the number of the SVRs needed to be computed is 18 (pointed by solid arrows). In fact, the dimension curse occurs only in the extreme case when all tracks are in the same neighborhood and with incomplete coverage. In this cases, when tracking *T* targets the number of SVRs would be 2^*T*^ − 1. For example, in the up left of Figure 3 the joint validation consists of three elliptical validation regions (centered at ${\hat{z}}^{1}$, ${\hat{z}}^{2}$, and ${\hat{z}}^{3}$, resp.). The total number of the SVRs is 2^3^ − 1 = 7 including one empty SVR (*n*_13_ = 0), so that the number of the SVRs needed to be computed is 6 (*n*_1_, *n*_2_, *n*_3_, *n*_12_, *n*_23_, and *n*_123_).

### 3.4. RBMCDA Algorithm with Gating Technique

The gating technique based RBMCDA algorithm is presented in Algorithm 1. This algorithm includes four major parts: prediction, finding validation regions and calculating *P*_*e*_*r*__^(*j*)^, calculating data association probabilities and updating the state of particles, and finally a particle weights calculation step. It should be pointed out that only the second part is fundamentally different from the original RBMCDA algorithm. In this algorithm, the target state priors can be represented as a weighted importance samples set

(17) $$\begin{array}{c} p\left(x_{0}^{\left(j\right)}\right)=\sum_{i=1}^{N_{p}}w_{i,0}𝒩\left(x_{0}^{\left(j\right)}\;|\;x_{i,0}^{\left(j\right)},P_{i,0}^{\left(j\right)}\right), \end{array}$$

where *i* is the identifier of particle with *N*_*p*_ being the number of particles employed. The dynamics and measurements for target *j* (*j* = 1,…, *T*) are assumed to be linear Gaussian

(18) $$\begin{array}{c} p\left(x_{k}^{\left(j\right)}\;|\;x_{k-1}^{\left(j\right)}\right)=𝒩\left(x_{k}^{\left(j\right)}\;|\;F_{k-1}^{\left(j\right)}x_{k-1}^{\left(j\right)},Q_{k-1}^{\left(j\right)}\right),\\ p\left(z_{k}\;|\;x_{k}^{\left(j\right)},c_{k}=j\right)=𝒩\left(z_{k}\;|\;H_{k}^{\left(j\right)}x_{k}^{\left(j\right)},R_{k}^{\left(j\right)}\right). \end{array}$$

## 4. Numerical Examples and Simulation Results

### 4.1. Numerical Examples

In some extreme cases (such as that all elliptical validation regions are separated, or coincide exactly), the *P*_*e*_^(*j*)^ obtained by different gating techniques will have the same value as *P*_*e*_^(*j*)^ = *P*_*g*_^(*j*)^ *P*_*d*_^(*j*)^.

This section just concerns with the general cases. Since *P*_*g*_^(*j*)^ is very close to unity (see Section 2.2), the results illustrated in the tables use *P*_*g*_^(*j*)^ = 1 (*j* = 1,…, *T*) for convenience. Without loss of generality, these examples do not consider the missed detection and assume *P*_*d*_^(*j*)^ = 1. In these examples, the clutter is modeled as independent and identically distributed with uniform spatial distribution. Examples 1 and 2 are about tracking two targets; Example 3 is about tracking three targets.

**Example 1**

**Figure 4:**
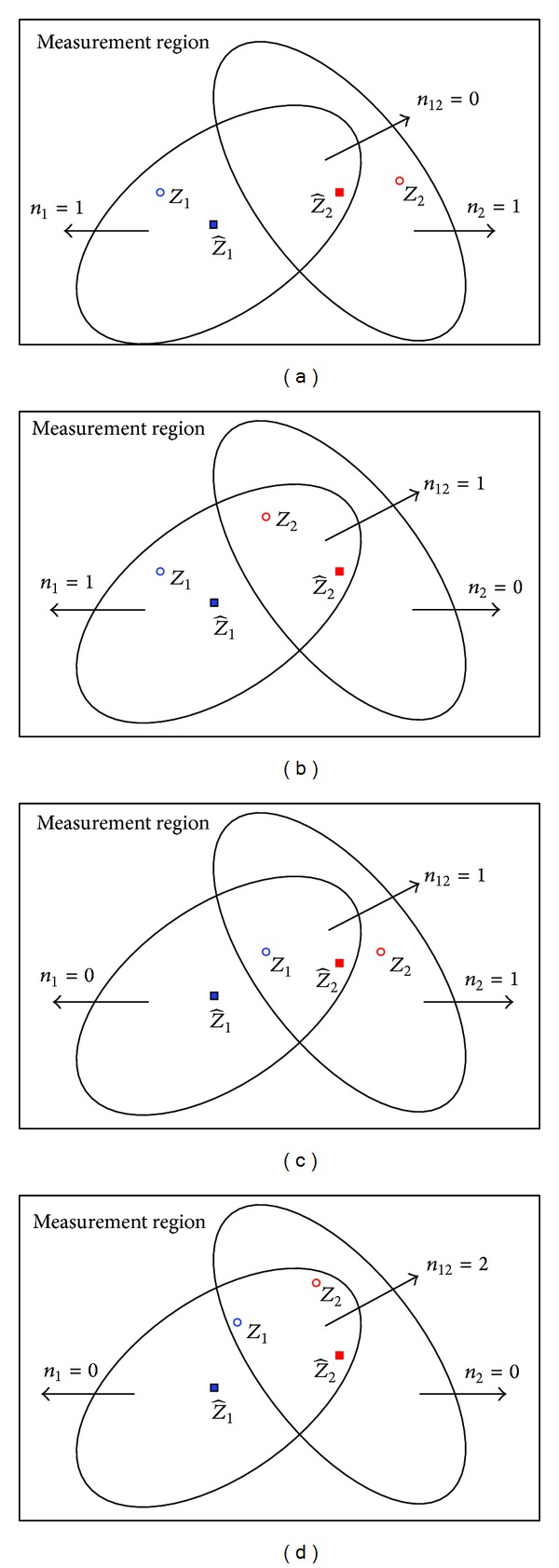
All possible hypotheses for measurements that fall inside two validation regions on condition that *P*_*d*_ = 1.

As shown in Figure 4, the numbers of the validated measurements in three SVRs will have four possible cases if clutter does not exist, (a) *n*_1_ = 1, *n*_2_ = 1, *n*_12_ = 0, (b) *n*_1_ = 1, *n*_2_ = 0, *n*_12_ = 1, (c) *n*_1_ = 0, *n*_2_ = 1, *n*_12_ = 1, and (d) *n*_1_ = 0, *n*_2_ = 0, *n*_12_ = 2.  Table 1 gives the *P*_*e*_*r*__^(*j*)^ obtained by different gating techniques, where “O-" represents the original RBMCDA and “U-," “J-," and “SJ-" represent the “U-RBMCDA,” “J-RBMCDA,” and “SJ-RBMCDA" algorithms, respectively. The results denoted as “-” mean that the corresponding subregions do not consist of any measurement. In this example, “O-” and “U-” have the same results because both of them do not consider the interrelations of the validated measurements. It can be seen that only the “J-” gives the correct results as expected. As a simplified version of the “J-,” the “SJ-” gives the trade-off results.

**Example 2**

In cluttered environment (see Figure 1(b)), two cases are studied, (e) *n*_1_ = 1, *n*_2_ = 1, *n*_12_ = 3 and (f) *n*_1_ = 1, *n*_2_ = 8, *n*_12_ = 2. In this example, “J-" and “SJ-" can give the similar results as shown in Table 2. Note that, “U-" still gives the result *P*_*e*_*r*__^(*j*)^ = 1 in each SVR *r*.

**Example 3**

**Figure 5:**
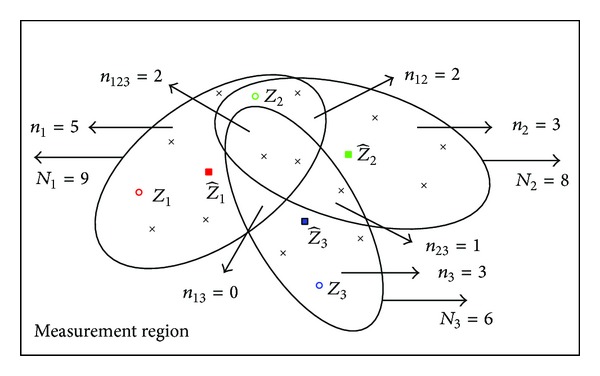
Example of measurement validation regions for tracking three targets.

In Figure 5, the numbers of validated measurements in three elliptical regions are *N*_1_ = 9, *N*_2_ = 8, and *N*_3_ = 6, respectively. The numbers of validated measurements in the SVRs are (g) *n*_1_ = 5, *n*_2_ = 3, *n*_3_ = 3, *n*_12_ = 2, *n*_13_ = 0, *n*_23_ = 1, and *n*_123_ = 2.  Table 3 gives the *P*_*e*_*r*__^(*j*)^ obtained by “J-" and “SJ-." In this three-target example, the “J-" and “SJ-" can also give the similar results. Next, we will give tracking examples to verify the performance of the algorithms.

### 4.2. Simulation Results

**Figure 6:**
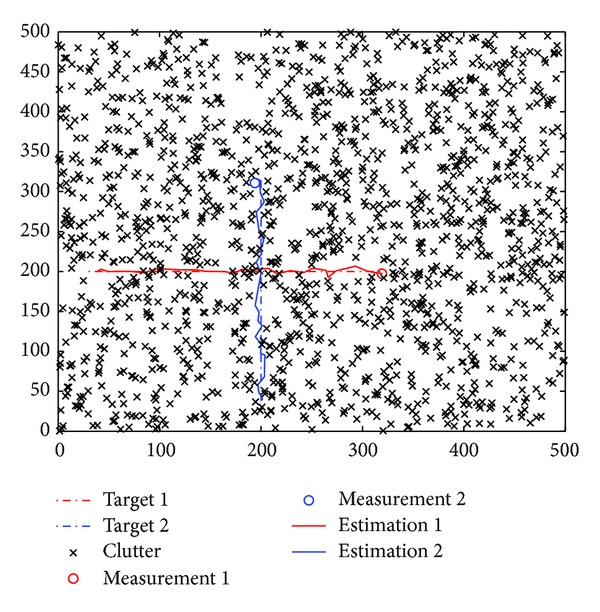
Example of tracking two crossing targets.

**Figure 7:**
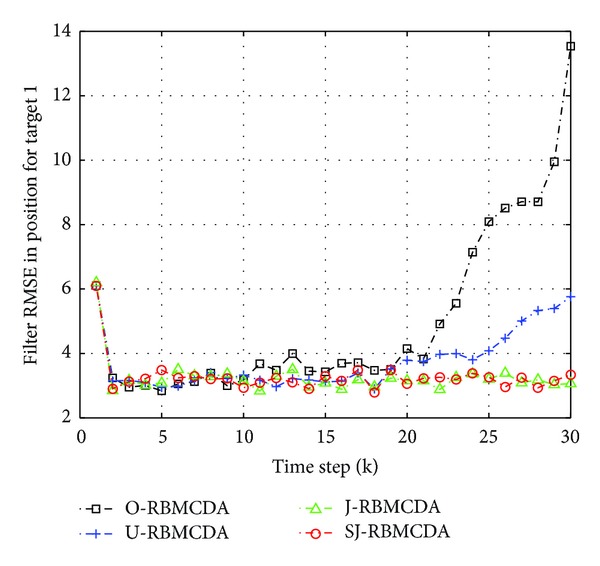
The average position estimation errors for target 1.

**Figure 8:**
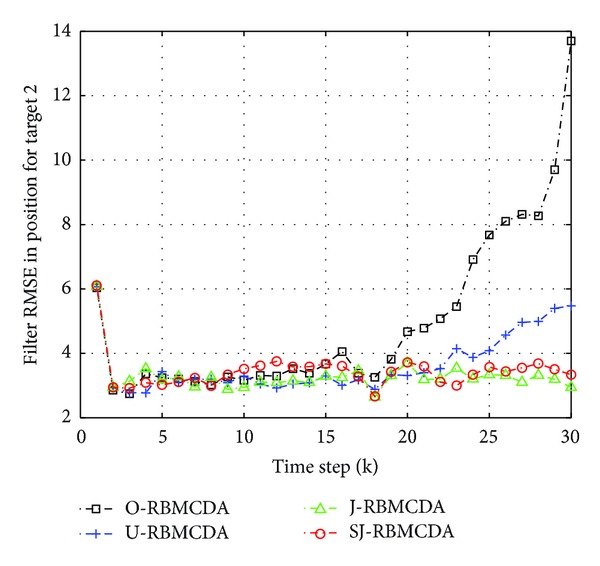
The average position estimation errors for target 2.

In the simulations, we model each target with constant velocity model in 2-dimensional Cartesian coordinates. The discrete-time state dynamic and measurement models for targets are given by (18), where

(19) $$\begin{array}{c} F_{k-1}^{\left(j\right)}=\left(\begin{array}{cccc} 1 & 0 & \Delta & 0\\ 0 & 1 & 0 & \Delta \\ 0 & 0 & 1 & 0\\ 0 & 0 & 0 & 1 \end{array}\right),\\ H_{k}^{\left(j\right)}=\left(\begin{array}{cccc} 1 & 0 & 0 & 0\\ 0 & 1 & 0 & 0 \end{array}\right),\\ G={\left(\begin{array}{cccc} \frac{1}{2}\Delta^{2} & 0 & \Delta & 0\\ 0 & \frac{1}{2}\Delta^{2} & 0 & \Delta \end{array}\right)}^{\prime },\\ Q_{k-1}^{\left(j\right)}=GG^{\prime } \end{array}$$

with the sample interval Δ = 1. The clutter is modeled as independent and identically distributed with uniform spatial distribution in a rectangular region of the coordinate plane, [0,500]×[0,500]. The number of clutter measurements obeys a Poisson distribution with the Poisson random number *λ* (clutter rate). Three tracking examples are studied to illustrate the performance and efficiency of the proposed gating techniques based RBMCDA algorithms. The first one involves a situation where two targets are crossing as shown in Figure 6. We want to compare the performance of the algorithms by the tracking position errors. The total number of time steps is set to be 30, the number of particles *N*_*p*_ = 100, the Poisson number *λ* = 50, the measurement variance **R**_*k*_^(*j*)^ = diag⁡  ([20,20]), and the detection probability *P*_*d*_^(*j*)^ = 0.99 (*j* = 1,…, 2). Figures 7 and 8 show the average root mean square errors (RMSE) of the four algorithms from 100 different runs. Here, the RMSE is referring to the average position estimation error. At time step *k*, the RMSE of the *j*th target is given by

(20) $$\begin{array}{c} \sqrt{\frac{1}{100}\sum_{j=1}^{2}\;\sum_{mc=1}^{100}{\left({\hat{x}}_{j,k}^{\left(m_{c}\right)}-x_{j,k}\right)}^{2}+{\left({\hat{y}}_{j,k}^{\left(m_{c}\right)}-y_{j,k}\right)}^{2}}, \end{array}$$

where $\left({\hat{x}}_{j,k}^{\left(m_{c}\right)},{\hat{y}}_{j,k}^{\left(m_{c}\right)}\right)$ are the corresponding position estimation in the *m*_*c*_th Monte Carlo run.

The simulation results show that the tracking process can be divided into two stages: before and after the happening of target crossing. In the first stage, the RMSE curves by all the algorithms have the similar values. It indicates that the proposed gating methods do not have an impact on the performance of the RBMCDA algorithm for tracking separated targets. In the second half of the tracking process, the significant arising of the RMSE curves by “O-" and “U-" algorithms indicates that, when target crossing happens, the “O-" and “U-" algorithms have more times of tracking failures (or mistracking) than “S-" and “SJ-" algorithms. This is because “S-" and “SJ-" can provide more accurate targets' origins than the “O-" and “U-" algorithms. In this example, the performance of “SJ-" is approximate to that of the “J-." This also supports the results obtained in the previous numerical examples.

**Figure 9:**
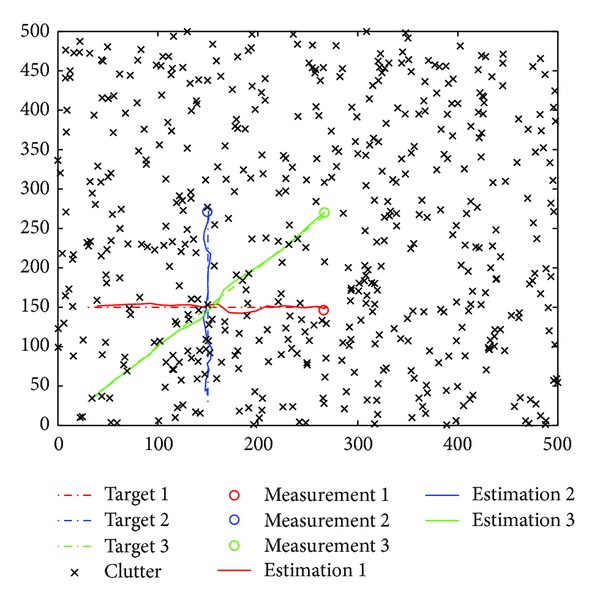
Example of tracking three crossing targets.

In the second example, we consider a three-target-crossing situation (as shown in Figure 9) to compare the time costs of the algorithms. The total number of time steps is set to be 25, the measurement variance **R**_*k*_^(*j*)^ = diag⁡  ([10,10]), and the detection probability *P*_*d*_^(*j*)^ = 0.8. We repeat each experiment 100 times and the average running times are listed in Tables 4 and 5, where we set a fixed clutter rate *λ* = 20 and a fixed number of particles *N*_*p*_ = 20. All simulations are performed on a PC with a 2.8-GHz Intel processor.

In Table 4, for all the four algorithms, the increases in time cost are proportional to the increases in the number of particles. In Table 5, only the computational time of “O-" is increased apparently with the clutter rate *λ*. This is because all added clutter should be considered in the data association procedures without using gating technique. The time costs of the proposed gating based algorithms grow moderately because only a small number of the added clutters can fall into the validation regions.

In both Tables 4 and 5, we can find that “U-" is the most efficient one. In the three gating based algorithms, “J-" needs the most computational time for its dealing with the joint events. Since “SJ-" uses a simple but more direct way, it needs less computational time than that of “J-." From the two tables, the gating technique based RBMCDA algorithms are much more efficient than the original RBMCDA algorithm because of the reduction of validated measurements. The relative efficiencies between these gating techniques can be summarized by “U-" > “SJ-" > “J-." In practice, although “U-" needs less computational time than “J-" and “SJ," but “U-" is not suitable for the use to track multiple closely spaced targets. In this situation, both the “J-" and “SJ-" can yield desirable results because of the rational consideration of the intersection regions.

## 5. Conclusion

This paper has studied the gating techniques in the application of the RBMCDA filter to multitarget tracking. Three different gating methods are presented and compared by computing both the tracking errors and time cost. “U-" incorporates a very simple gate into the framework of the RBMCDA approach. “J-" can take the measurement uncertainties into account reasonably by calculating the joint events. As a simplification of “J," “SJ-" is more efficient than “J" and the tracking performance loss incurred is not significant in the experimental examples. Therefore, “SJ-" can be a time-saving choice for practical applications. It should be pointed out that the gating techniques discussed in this paper may eliminate all measurements outside the validation regions of the targets that have already been detected, which will make it difficult to detect newly appearing targets. To address this, the proposed gating techniques could be started after the initialization periods of the RBMCDA filter to avoid impairing the tracks initiation.

## Figures and Tables

**Algorithm 1:**
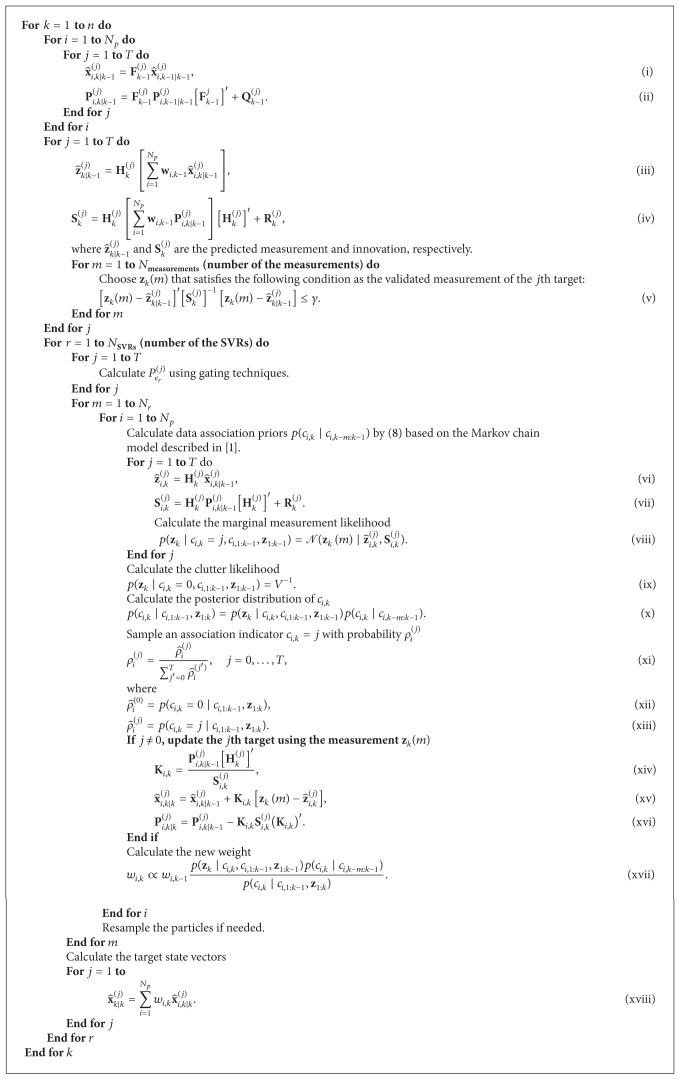
RBMCDA using gating techniques.

**Table 1:** The *P*_*e*_*r*__^(*j*)^ obtained by four different methods: two-target situation.

(*a*)/(*b*)/(*c*)/(*d*)	*P*_*e*_1__^(1)^	*P*_*e*_2__^(2)^	*P*_*e*_12__^(1)^	*P*_*e*_12__^(2)^
O-	1/1/—/—	1/1/—/—	1/1/—/—	1/0.5/—/—
U-	1/—/1/—	1/—/1/—	1/—/1/—	1/—/0.5/—
J-	—/1/1/1	—/1/1/1	—/0/1/1	—/0.5/1/1
SJ-	—/1/1/1	—/1/1/1	—/1/0/1	—/1/0.5/1

**Table 2:** The *P*_*e*_*r*__^(*j*)^ obtained by “U”, “J-”, and “SJ-”: two-target situation.

(*e*)/(*f*)	*P*_*e*_1__^(1)^	*P*_*e*_2__^(2)^	*P*_*e*_12__^(1)^	*P*_*e*_12__^(2)^
U-	1/1	1/1	1/1	1/1
J-	0.3077/0.1220	0.3077/0.2195	0.6923/0.8780	0.6923/0.7805
SJ-	0.25/0.1111	0.25/0.2	0.75/0.8889	0.75/0.8

**Table 3:** The *P*_*e*_*r*__^(*j*)^ obtained by “J-” and “SJ-”: three-target situation.

(*g*)	*P*_*e*_1__^(1)^	*P*_*e*_2__^(2)^	*P*_*e*_3__^(3)^	*P*_*e*_12__^(1)^	*P*_*e*_12__^(2)^	*P*_*e*_23__^(2)^	*P*_*e*_23__^(3)^	*P*_*e*_123__^(1)^	*P*_*e*_123__^(2)^	*P*_*e*_123__^(3)^
J-	0.61	0.42	0.55	0.21	0.25	0.12	0.16	0.18	0.21	0.29
SJ-	0.56	0.38	0.5	0.22	0.25	0.13	0.17	0.22	0.25	0.33

**Table 4:** Computational time (sec) using different numbers of particles.

Numbers of particles	20	40	60	80	100
O-	12.1373	18.2593	25.3149	31.7860	38.4171
U-	1.1085	2.2064	3.3344	4.4614	5.6046
J-	1.2446	2.5641	4.0014	5.3252	6.5214
SJ-	1.1248	2.3910	3.6465	4.8110	5.8696

**Table 5:** Computational time (sec) in different clutter rates.

Clutter rates (*λ*)	20	40	60	80	100
O-	12.0975	17.5701	22.9209	28.4144	34.0663
U-	1.0522	1.1306	1.2546	1.3155	1.3967
J-	1.1965	1.2832	1.4081	1.4914	1.6091
SJ-	1.0925	1.2024	1.3330	1.4350	1.5127
